# Age and gender differences of basic electrocardiographic values and abnormalities in the general adult population; Tehran Cohort Study

**DOI:** 10.1186/s12872-023-03339-z

**Published:** 2023-06-16

**Authors:** Pooria Ahmadi, Arian Afzalian, Arash Jalali, Saeed Sadeghian, Farzad Masoudkabir, Alireza Oraii, Aryan Ayati, Sepehr Nayebirad, Parmida Sadat Pezeshki, Masoumeh Lotfi Tokaldani, Akbar Shafiee, Mohammad Mohammadi, Elham Sanei, Masih Tajdini, Kaveh Hosseini

**Affiliations:** 1 Tehran Heart Center, Cardiovascular Diseases Research Institute, Tehran University of Medical Sciences, Tehran, Iran; 2 Cardiac Primary Prevention Research Center, Cardiovascular Diseases Research Institute, Tehran University of Medical Sciences, Tehran, Iran

**Keywords:** Electrocardiography, Arrhythmia, ECG abnormalities, Epidemiology, Iran, Age distribution, Sex distribution

## Abstract

**Background:**

Although several studies are available regarding baseline Electrocardiographic (ECG) parameters and major and minor ECG abnormalities, there is considerable controversy regarding their age and gender differences in the literature.

**Methods:**

Data from 7630 adults aged ≥ 35 from the Tehran Cohort Study registered between March 2016 and March 2019 were collected. Basic ECG parameters values and abnormalities related to arrhythmia, defined according to the American Heart Association definitions, were analyzed and compared between genders and four distinct age groups. The odds ratio of having any major ECG abnormality between men and women, stratified by age, was calculated.

**Results:**

The average age was 53.6 (± 12.66), and women made up 54.2% (*n* = 4132) of subjects. The average heart rate (HR) was higher among women(*p* < 0.0001), while the average values of QRS duration, P wave duration, and RR intervals were higher among men(*p* < 0.0001). Major ECG abnormalities were observed in 2.9% of the study population (right bundle branch block, left bundle branch block, and Atrial Fibrillation were the most common) and were more prevalent among men compared to women but without statistical significance (3.1% vs. 2.7% *p* = 0.188). Moreover, minor abnormalities were observed in 25.9% of the study population and again were more prevalent among men (36.4% vs. 17% *p* < 0.001). The prevalence of major ECG abnormalities was significantly higher in participants older than 65.

**Conclusion:**

Major and minor ECG abnormalities were roughly more prevalent in male subjects. In both genders, the odds of having major ECG abnormalities surge with an increase in age.

## Introduction

Cardiovascular diseases (CVDs) are the leading causes of death globally [1, 2]. Screening and early diagnosis of CVDs are crucial in identifying high-risk cases and developing preventive strategies for minimizing mortality rates worldwide. Electrocardiography (ECG) is a global, inexpensive, non-invasive, and easily-accessible technique for screening and diagnosing heart diseases. Although several studies are available about baseline ECG parameters and major or minor ECG abnormalities, there is considerable controversy regarding sex and age differences.

Age, sex, and ethnicity are important contributors to variations in ECG values and abnormalities [3–5]. Some of the main Studies implied a better interpretation of ECG based on the aforementioned variables [6–8]. These discrepancies are also bolded when comparing different countries and races. However, limited data are available regarding the age and gender effect on ECG variations in Middle Eastern Countries such as Iran. Similar to the worldwide data, cardiovascular diseases are the leading cause of morbidity and mortality in Iran [9, 10]. Therefore understanding age and gender disparities in ECG abnormalities and values is crucial for improving the accuracy of ECG interpretation and optimizing cardiovascular risk assessment in diverse populations. In this study, we aimed to report the prevalence of major and minor baseline ECG abnormalities, especially concerning arrhythmia, in the general population of Tehran (the capital of Iran). In addition, average ECG parameters and arrhythmias were compared separately in sex and age groups.

## Material and methods

### Study design, participants, and setting

In this study, we extracted data from the Tehran cohort study (TeCS), a prospective population-based multidisciplinary longitudinal study conducted among adults residing in Tehran, the capital of Iran. The primary aim was to provide a descriptive analysis of the prevalence of ECG abnormalities based on age and gender groups. Study (TeCS) protocol has been published previously [11].

Ten thousand households from 22 districts of Tehran were selected via a systematic sampling method between March 2016 to March 2019. The inclusion criteria comprised being a permanent resident of Tehran and having at least one member older than 35 in the household. A final number of 4215 (42.1%) households participated in the study resulting in 8296 cardiovascular evaluations from the participating families, and their personal, clinical, and para-clinical data were collected. Several questionnaires were utilized to determine and assess the participants' demographic characteristics, habitual risk factors, medical/medication history, psychological health, and injury occurrence. Participants assessments wer performed at Tehran Heart Center which is a tertiary cardiovascular hospital affiliated with the Tehran University of Medical Sciences. Informed consent was obtained from all subjects and/or their legal guardian(s).The TeCS protocol has been approved by the board of research and the committee of medical ethics at Tehran University of Medical Sciences (code of ethics: IR.TUMS.MEDICINE.REC.1399.074).

### Resting ECG measurement

Twelve‑lead ECG was obtained using a 12-channel M-TRACE ECG device (M4Medical, Lublin, Poland), according to standard methods and instructions for lead placement. We archived a printed copy of the ECG in the participants' files and sent them another transcript in their report package. Also, the electronic ECG documents were saved on the TeCS server. Finally, the ECG records were reviewed and interpreted by two expert cardiologists (P.A and K.H) and re-evaluated by the third cardiologist (M.T) in case of discrepancies.

### Definitions of ECG abnormalities

Following the diagnosis of an ECG abnormality, the ECG print was reviewed by two other expert cardiologists. ECG abnormality definitions were performed based on a similar prior article [12]. The ECG arrhythmias were then categorized as minor or major based on whether they were common, clinically irrelevant, or clinically important and relevant, respectively.

The major ECG abnormalities comprised of Atrial Fibrillation (AF), Atrial Flutter (AFL), left bundle branch block (LBBB), right bundle branch block (RBBB), second-degree atrioventricular block (AVB), complete heart block (CHB), Wolff-Parkinson-White (WPW), supraventricular or ventricular tachycardia, and artificial pacemaker. Also, the following ECG findings, sinus bradycardia, premature junctional rhythm (PJC), premature ventricular rhythm (PVC), premature atrial rhythm (PAC), first-degree AVB, incomplete RBBB, incomplete LBBB, left anterior fascicular hemiblock (LAFB) and left posterior fascicular hemiblock (LPFB) were included in the minor abnormalities group. Apart from the abnormalities mentioned above, some channelopathies were also evaluated, Brugada pattern and repolarization changes, including anterior early repolarization and inferior/inferolateral early repolarization, were also documented. Finally, left ventricular hypertrophy was defined according to Sokolow-Lyon criteria [13]. Also, left atrial hypertrophy(enlargement) and left, right, or extreme right axis deviation were documented and analyzed. Finally, MI patterns, including Q wave, T wave Inversion, and ST segment changes, were defined according to AHA recommendations [12, 14].

### Statistical analysis

Normally distributed continuous variables, including age, Systolic Blood Pressure (SBP), Diastolic Blood Pressure (DBP), High-Density Lipoprotein (HDL), total cholesterol, RR interval, Heart Rate (HR), max QRS duration (QRSd), and P wave duration, were reported as mean with standard deviation. Meanwhile, Triglyceride (TG) and Low-Density Lipoprotein (LDL) were skewed distributed, so they were described as median with 25th and 75th percentiles. Normally distributed variables were compared between males and females using an independent sample's t-test; these variables were compared between age categories using a one-way analysis of variance (ANOVA). Categorical variables, ECG characteristics, were compared between men and women or age categories using chi-squared or Fisher's exact test, as appropriate. The association of age categories with the persistence of major components of ECG was evaluated by applying the Logistic regression model and was reported through odds ratio (OR) with a 95% confidence interval (CI). All statistical analyses were conducted using IBM SPSS Statistics for Windows, version 23.0 (Armonk, NY: IBM Corp).

## Results

### Participants

ECG information of 7630 participants was available. The study population's average age was 53.6 (± 12.66). 54.2% of the study population were women (*n* = 4132), and 45.8% (*n* = 3498) were men. Table 1 illustrates the baseline characteristics of 7630 participants in the TeCS.

**Table 1:** Baseline characteristics of 7630 participants

	**Total (7630)**	**F** **(4132)**	**M** **(3498)**	***p*-value**	**35–44 (2173)**	**45–54 (2053)**	**55–64 (1796)**	** > 65 (1608)**	***p*-value**
**F**	4132 (54.2%)	-	-	-	1240 (57.1%)	1120 (54.6%)	1016 (56.6%)	756 (47.0%)	< 0.001
**M**	3498 (45.8%)	-	-	-	933 (42.9%)	933 (45.4%)	780 (43.4%)	852 (53.0%)	< 0.001
**Age**	53.6 ± 12.66	52.7 ± 12.23	54.6 ± 13.07	< 0.001	39.1 ± 2.89	49.4 ± 2.84	59.1 ± 2.82	72.3 ± 6.46	-

Data are presented as number (percentage), mean ± standard deviation, or median [25th—75th]

*Abbreviations*: *F* Female, *M* Male

### ECG basic values

**Fig. 1:**
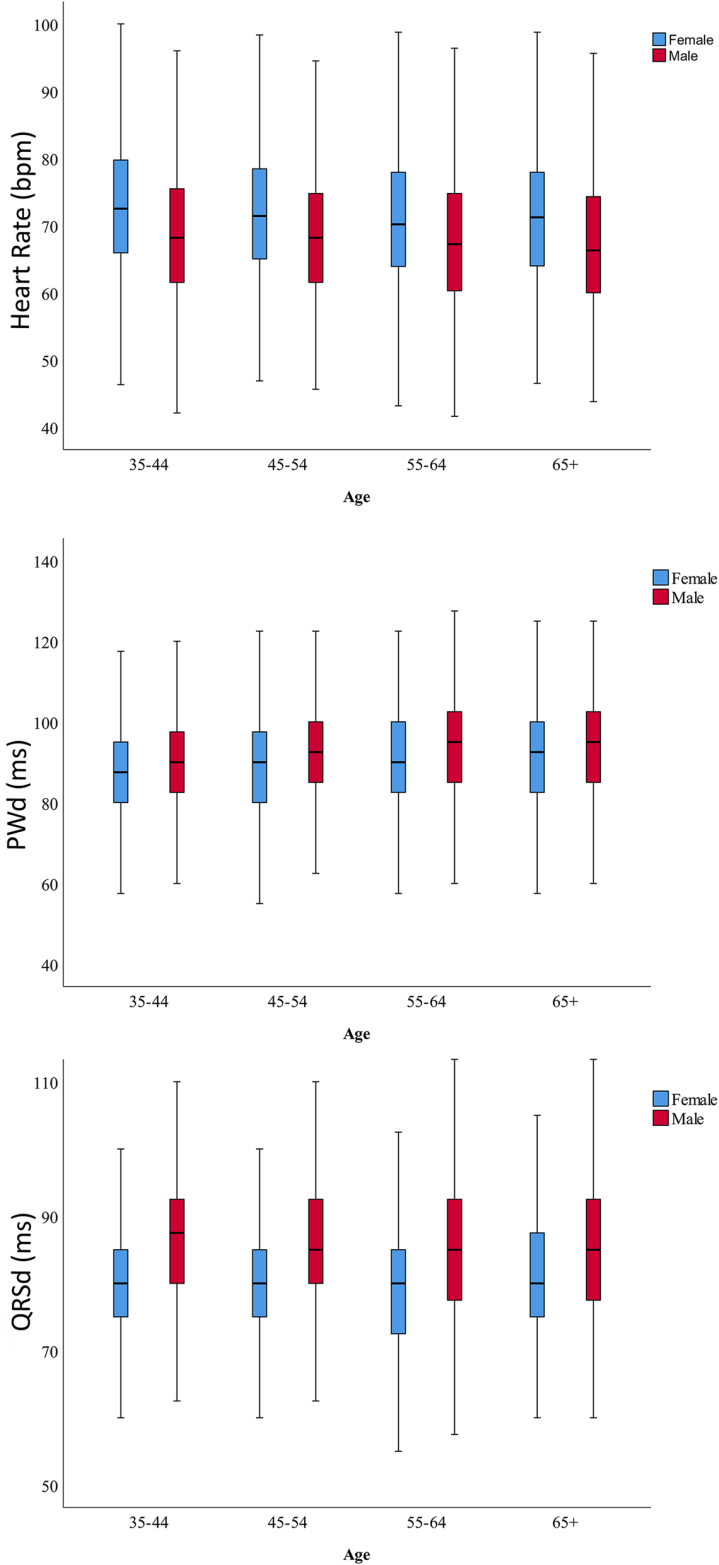
Average ECG basic values in Tehran general population, stratified by age and gender. QRSd, QRS Duration; PWd, P Wave duration; HR, Heart Rate

The mean HR was 70.6 (± 11.03) bpm. Also, the average RR interval, QRSd, and P-wave duration were 870.6 ms, 83.9 ms, and 90.7 ms, respectively. In addition, the mean HR was measured significantly higher among females than males (72.4 vs. 68.4 *p* < 0.001). However, the average values of the following ECG parameters were assessed to be significantly higher among men compared to women: RR interval (897.8 vs. 847.3 *p* < 0.001), QRSd (86.8 vs. 81.3 *p* < 0.001), and P-wave duration (92.5 vs. 89.2 *p* < 0.001). The thorough details of the prevalence and comparison of ECG values between males and females are presented in Table 2. Moreover, Table 3 illustrates the average ECG parameters in all four age groups separated by gender. Figure 1 demonstrates the mean ECG values in all four age groups in both genders, as well.

**Table 2:** Prevalence of major and minor electrocardiographic abnormalities by gender

**ECG pattern**	**Total (%)** ***n* = 7630**	**F (%)** ***n* = 4132**	**M (%)** ***n* = 3498**	***p*-Value**
**Major abnormalities**				
** Total**	221(2.89)	111(2.68)	110 (3.14)	0.188
** AF**	40(0.52)	25 (0.6)	15 (0.4)	0.288
** AFL**	3(0.03)	1 (0)	2 (0.1)	0.597
** LBBB**	73(0.95)	47 (1.1)	26 (0.7)	0.078
** RBBB**	101(1.32)	37 (0.9)	64 (1.8)	< 0.001
** WPW**	4(0.05)	1 (0)	3 (0.1)	0.339
**Minor abnormalities**				
** Total**	1,973 (25.84)	702 (16.98)	1271 (36.33)	< 0.001
** Sinus Bradycardia**	1145(15)	412 (10.7)	733 (22.4)	< 0.001
** PVC**	95(1.24)	39 (0.9)	56 (1.6)	0.010
** PAC**	67(0.87)	29 (0.7)	38 (1.1)	0.073
** First-degree AVB**	70(0.91)	21 (0.5)	49 (1.4)	< 0.001
** Incomplete RBBB**	218(2.85)	73 (1.8)	145 (4.1)	< 0.001
** Incomplete LBBB**	1(0.01)	0 (0)	1 (0)	0.458
** LAFB**	345(4.52)	113 (2.7)	232 (6.6)	< 0.001
** LPFB**	32(0.41)	15 (0.4)	17 (0.5)	0.408
**QRS axis deviation**				
** Total**	464(6.08)	167(2.18)	297(3.89)	< 0.001
** Left deviation**	394(5.16)	137 (3.3)	257 (7.3)	< 0.001
** Right deviation**	63(0.82)	29 (0.7)	34 (1)	< 0.001
** Extreme Right deviation**	7(0.09)	1 (0)	6 (0.2)	< 0.001
**Chamber enlargement patterns**				
** LVH**	244(3.19)	69(1.7)	175(5)	< 0.001
** LAE**	360(4.71)	108 (2.6)	252(7.2)	< 0.001
**Brugada, and early repolarization**				
** Brugada Pattern**	21(0.27)	1 (0)	20 (0.6)	< 0.001
** Anterior Early repolarization**	37(0.48)	4 (0.1)	33(0.9)	< 0.001
** Inferior/Inferolateral Early repolarization**	119(1.42)	20 (0.5)	89 (2.5)	< 0.001
** Sinus Arrhythmia**	22(0.28)	12 (0.3)	10 (0.3)	0.971
**ECG values**				
** RR interval (ms)**	870.6 ± 133.18	847.3 ± 124.6	897.8 ± 137.7	< 0.001
** HR (bpm)**	70.6 ± 11.03	72.4 ± 10.95	68.4 ± 10.73	< 0.001
** QRS duration (ms)**	83.9 ± 12.77	81.3 ± 11.72	86.8 ± 13.31	< 0.001
** P-wave duration (ms)**	90.7 ± 12.55	89.2 ± 12.2	92.5 ± 12.72	< 0.001
**MI Patterns**				
** ST segment depression**	430(5.63)	288 (7%)	142 (4.1%)	< 0.001
** T wave inversion**	600(7.9%)	365 (8.8%)	235 (6.7%)	0.001
** Q wave**	141(1.8)	43 (1.0%)	98(2.8%)	< 0.001

Data are presented as numbers (percentage) and mean ± standard deviation

***Abbreviations:**** AF* Atrial fibrillation, *AFL* Atrial flutter, *LBBB* Left bundle branch block, *RBBB* Right bundle branch block, *WPW* Wolf-Parkinson-White, *PVC* Premature ventricular complex, *PAC* Premature atrial complex, *AVB* Atrioventricular block, *LAFB* Left anterior fascicular block, *LPFB* Left posterior fascicular block, *LVH* Left ventricular hypertrophy, *LAE* Left atrial enlargement, *F-QRS* Fragmented QRS, *HR* Heart rate

**Table 3:** Prevalence of major and minor electrocardiographic abnormalities stratified by age and groups

**Age Group**		**35–44**			**45–54**			**55–64**			** > 65**		
**ECG parameters**	**Total (%)** ***n* = 7630**	**F (%)** ***n* = 1240**	**M (%)** ***n* = 933**	***p*-value**	**F (%)** ***n* = 1120**	**M (%)** ***n* = 933**	***p*-value**	**F (%)** ***n* = 1016**	**M (%)** ***n* = 780**	***p*-value**	**F (%)** ***n* = 756**	**M (%)** ***n* = 852**	***p*-value**
**Major abnormalities**													
Total	221 (2.89)	8 (0.6)	5 (0.5)	0.929	10 (0.9)	5 (0.5)	0.143	29 (2.9)	15 (1.9)	0.206	64(8.5)	85 (10.0)	0.184
AF	40 (0.52)	1 (0.1)	0 (0)	> 0.999	3 (0.3)	0 (0)	0.256	9 (0.9)	1 (0.1)	0.05	12 (1.6)	14 (1.6)	0.929
AFL	3 (0.03)	0 (0)	0 (0)	-	0 (0)	0 (0)	-	1 (0.1)	0 (0)	> 0.999	0 (0)	2 (0.2)	0.501
LBBB	73 (0.95)	2 (0.2)	1 (0.1)	> 0.999	3 (0.3)	0 (0)	0.256	9 (0.9)	3 (0.4)	0.196	33 (4.4)	22 (2.6)	0.05
RBBB	101 (1.32)	5 (0.4)	3 (0.3)	> 0.999	3 (0.3)	4 (0.4)	0.709	10 (1)	11 (1.4)	0.405	19 (2.5)	46 (5.4)	0.003
WPW	4 (0.05)	0 (0)	1 (0.1)	0.429	1 (0.1)	1 (0.1)	> 0.999	0 (0)	0 (0)	-	0 (0)	1 (0.1)	> 0.999
**Minor abnormalities**													
Total	1,973 (25.84)	130 (10.5)	259 (27.7)	< 0.001	168 (15)	301 (32.26)	< 0.001	192 (18.89)	294 (37.69)	< 0.001	212 (28)	417 (48.94)	< 0.001
Sinus Bradycardia	1145 (15)	86 (7.3)	179 (20.1)	< 0.001	114 (11)	177 (20.2)	< 0.001	116 (12.4)	177 (24.3)	< 0.001	96 (13.9)	200 (25.7)	< 0.001
PVC	95 (1.24)	6 (0.5)	2 (0.2)	0.479	9 (0.8)	11 (1.2)	0.388	14 (1.4)	10 (1.3)	0.861	10 (1.3)	33 (3.9)	0.002
PAC	67 (0.87)	3 (0.2)	0 (0)	0.265	3 (0.3)	4 (0.4)	0.709	6 (0.6)	7 (0.9)	0.447	17 (2.2)	27 (3.2)	0.259
First-degree AV block	70 (0.91)	2 (0.2)	4 (0.4)	0.412	4 (0.4)	5 (0.5)	0.74	3 (0.3)	10 (1.3)	0.014	12 (1.6)	30 (3.5)	0.015
Incomplete RBBB	218 (2.85)	14 (1.1)	42 (4.5)	< 0.001	16 (1.4)	49 (5.3)	< 0.001	20 (2)	32 (4.1)	0.008	23 (3)	22 (2.6)	0.577
Incomplete LBBB	1 (0.01)	0 (0)	0 (0)	-	0 (0)	0 (0)	-	0 (0)	0 (0)	-	0 (0)	1 (0.1)	> 0.999
LAFB	345 (4.52)	12 (1)	23 (2.5)	0.006	20 (1.8)	52 (5.6)	< 0.001	31 (3.1)	55 (7.1)	< 0.001	50 (6.6)	102 (12)	< 0.001
LPFB	32 (0.41)	7 (0.6)	9 (1)	0.28	2 (0.2)	3 (0.3)	0.664	2 (0.2)	3 (0.4)	0.658	4 (0.5)	2 (0.2)	0.429
**QRS axis deviation**													
Left deviation	394 (5.16)	13 (1)	21 (2.3)	0.065	21 (1.9)	53 (5.7)	< 0.001	39 (3.8)	61 (7.8)	0.001	64 (8.5)	122 (14.3)	< 0.001
Right deviation	63 (0.82)	17 (1.4)	16 (1.7)	0.405	3 (0.3)	7 (0.8)	< 0.001	3 (0.3)	5 (0.6)	0.658	6 (0.8)	6 (0.7)	0.664
Extreme Right deviation	7 (0.09)	0 (0)	0 (0)	-	0 (0)	1 (0.1)	0.429	1 (0.1)	2 (0.3)	0.429	0 (0)	3 (0.4)	0.252
**Chamber enlargement patterns**													
LVH	244 (3.19)	8 (0.6)	35 (3.8)	< 0.001	10 (0.9)	38 (4.1)	< 0.001	16 (1.6)	38 (4.9)	< 0.001	35 (4.6)	64 (7.5)	0.016
LAE	360 (4.71)	3 (0.2)	20 (2.1)	< 0.001	18 (1.6)	55 (5.9)	< 0.001	35 (3.4)	72 (9.2)	< 0.001	52 (6.9)	105 (12.3)	< 0.001
**Brugada, and early repolarization**													
Brugada Pattern	21 (0.27)	0 (0)	6 (0.7)	0.006	0 (0)	4 (0.4)	0.042	0 (0)	5 (0.7)	0.016	1 (0.2)	5 (0.7)	0.222
Anterior Early repolarization	37 (0.48)	1 (0.1)	8 (0.9)	< 0.001	0 (0)	5 (0.5)	< 0.001	3 (0.3)	13 (1.7)	< 0.001	0 (0)	7 (0.8)	0.007
Inferior/Inferolateral Early repolarization	119 (1.42)	6 (0.5)	43 (4.6)	< 0.001	8 (0.7)	26 (2.8)	< 0.001	4 (0.4)	12 (1.5)	< 0.001	2 (0.3)	8 (0.9)	0.006
**ECG values**													
RR interval ± ms	870.6 ± 133.18	833.6 ± 117.39	890 ± 132.93	< 0.001	849 ± 122.44	890.7 ± 128.09	< 0.001	858.7 ± 126.26	904.5 ± 142.24	< 0.001	852.9 ± 135.14	908.4 ± 148.05	< 0.001
Heart Rate	70.6 ± 11.03	73.4 ± 10.67	68.9 ± 10.25	< 0.001	72.2 ± 10.67	68.8 ± 9.97	< 0.001	71.4 ± 10.82	68 ± 10.82	< 0.001	72.2 ± 11.85	67.9 ± 11.93	< 0.001
QRS duration ± ms	83.9 ± 12.77	80.9 ± 9.64	86.9 ± 10.02	< 0.001	80.5 ± 9.38	86.3 ± 10.37	< 0.001	80.8 ± 11.95	85.5 ± 12.51	< 0.001	83.8 ± 16.39	88.6 ± 18.76	< 0.001
P-wave duration ± ms	90.7 ± 12.55	87.2 ± 11.12	90.2 ± 11.5	< 0.001	88.9 ± 11.51	92 ± 12.01	< 0.001	90.5 ± 12.59	93.7 ± 13.2	< 0.001	91.1 ± 13.77	94.5 ± 13.85	< 0.001
**MI Patterns**													
ST segment depression	430 (5.63)	47 (3.8%)	14 (1.5%)	0.001	53 (4.7%)	22 (2.4%)	0.004	77 (7.6%)	23 (2.9%)	< 0.001	111 (14.7%)	83 (9.7%)	0.002
T wave inversion	600(7.9%)	4(0.3%)	6(0.6%)	0.30	9(0.8%)	17(1.8%)	0.002	12(1.2%)	23(2.9%)	0.026	18(2.4%)	52(6.1%)	0.058
Q wave	141(1.8)	65(5.2%)	40(4.3%)	0.34	91(8.1%)	44(4.7%)	0.40	96(9.4%)	51(6.5%)	0.007	113(14.9%)	100(11.7%)	< 0.001

Data are presented as numbers (percentage) and mean ± standard deviation

*Abbreviations*: *AF* Atrial fibrillation, *AFL* Atrial flutter, *LBBB* Left Bundle Branch Block, *RBBB* Right Bundle Branch Block, *PVC* Premature Ventricular Complex, *PAC* Premature Atrial Complex, *LAFB* Left Anterior Fascicular Block, *LPFB* Left Posterior Fascicular Block, *LVH* Left ventricular hypertrophy, *LAE* Left Atrial Enlargement, *F QRS* Fragmented QRS, *HR* Heart rate

### ECG Abnormalities

**Fig. 2:**
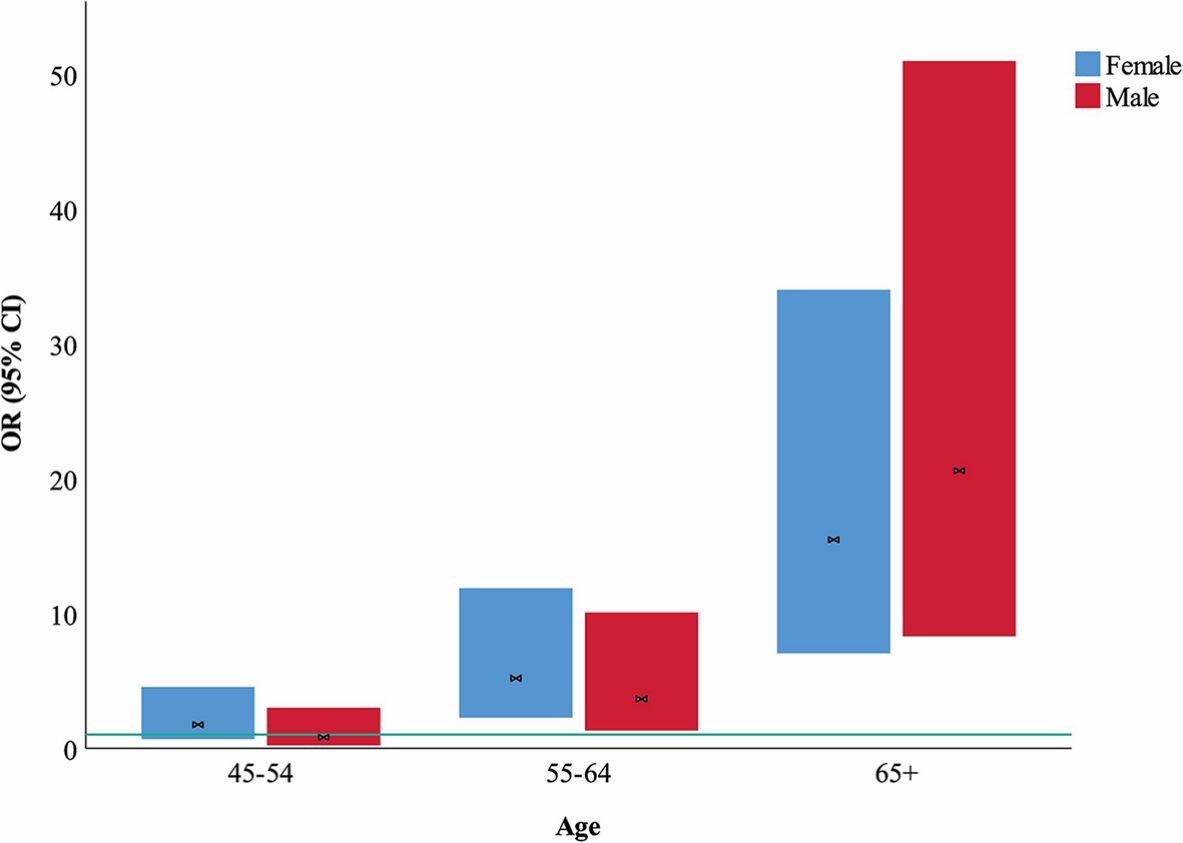
OR of having any major ECG abnormality among males and females stratified by age. The Bars illustrate the measured odds ratio (OR) and the 95% confidence interval (CI). The reference value for the age group is 34 to 45

Major ECG abnormalities were observed in 2.9% (*n* = 221) of the study population, with RBBB, LBBB, AF, and AFL being the most common, respectively. Major ECG abnormalities were more prevalent among males compared to females but statically did not have a significant *P*-value (3.1% vs. 2.7% *p* = 0.188). No cases of CHB or 2nd-degree AV block were observed. In addition, the odds of having major ECG abnormalities were associated with a higher age in both genders (Fig. 2). Minor abnormalities were also observed in 25.8% (*n* = 1973) of the study population. They were more prevalent among men than women (36.3% vs. 17% *p* < 0.001). Table 2 and Table 3 demonstrate the prevalence of major and minor electrocardiographic abnormalities among males and females and in four pre-defined age groups of the study population.

The prevalence of ECG abnormalities in our study was estimated to be 15% for sinus bradycardia, 0.56% for both AF and AFL, 2.28% for bundle branch blocks (both LBBB and RBBB), 2.12% for premature contractions (both PVC and PAC), 2.87% for incomplete bundle branch blocks (both incomplete LBBB, and RBBB), 0.91% for first degree AV block, and 4.94% for left fascicular hemiblocks (both LAFB and LPFB).

Regarding the heart (QRS) axis, 5.16%, 0.82%, and 0.09% of the study population had ECG patterns consistent with left, right, and extreme right QRS axis deviation, respectively. In addition, in terms of chamber abnormalities, left ventricular hypertrophy and left atrial hypertrophy(enlargement) patterns were observed in 3.19% and 4.71% of the participants, respectively.

Furthermore, the Brugada pattern, anterior early repolarization, and inferior/inferolateral early repolarization were observed in 0.27%, 0.48%, and 1.42% of the study population, respectively. Finally, MI patterns, including ST-depression, T wave inversion, and Q wave, were reported in 5.63, 7.9%, and 1.8% of the population, respectively. The prevalence of these patterns increased with age and was significantly more common among men.

## Discussion

Few data are available regarding age- and sex-related differences in ECG parameters. Thus, this study analyzed the average ECG values, abnormalities, and demographic features in a large sample size of Tehran residents. The average HR was higher among women, while the average values of QRSd, P wave duration, and RR intervals were higher among men. In addition, major and minor ECG abnormalities were observed in 2.9% and 25.9% of the study population, respectively, and both were more prevalent among men than women. However, these differences were not statistically significant in major abnormalities.

The etiologic causes of the observed ECG differences between age and gender groups are not fully understood. Nevertheless, several mechanisms might contribute to these differences. Variations of sex hormones [15], cardiac structure [16], CAD risk and presentations [17], body compositions [18], and cardiac pathology differences [19, 20] are some of the suggested contributors to ecg differences between men and women. Moreover, developmental, structural, and electrical conduction system changes in the heart during life can significantly contribute to ECG differences between different age groups [21, 22]. Additionally, variations in the risk of atherosclerosis and other CVDs [23, 24], coexisting diseases [25, 26], medications [27, 28], and lifestyle factors [29, 30] can substantially affect ECG components and abnormality across different ages.

### ECG basic values

#### HR

The average HR of the female participants in our study was higher compared to males, which was consistent with the findings of several previous studies [31–35]. The results of a large study enrolling 486,014 Brazilians demonstrated a decrease in the HR median with aging from the first year of life and then stabilization at 65-66 bpm in males compared to 70-73 bpm in females between 19 to 79 years of age [36]. Another similar study conducted among 14,424 adults also reported a significantly lower median HR of 64 for men compared to 67 for women [37].

### QRS and P wave durations

In contrast to HR, the average values of QRSd and P wave duration were assessed to be higher among men than women in our study. Previous studies have also reported the same gender differences, with higher QRSds among males [8, 32–35, 38].

In the first three age groups between ages 35 to 65, QRSd remained almost stable. However, it was prolonged in the > 65 years-old age group in both genders, specifically among women. Mason et al. observed stabilized mean QRSd values with increasing age [33]. Likewise, Rijnbeek et al. noted an almost constant QRSd for all ages [8]. The QRSd gender discrepancy can be interpreted because of comparatively smaller cardiac mass (particularly the left ventricle) in women.

P wave duration was also analyzed in this study, and it constantly rose with age in both genders and peaked in the > 65 age group. Consistent with our results, Rijnbeek et al. stated that the P and PR durations were longer for men compared to women and reported a slight, constant increase in both values with aging [8].

### QRS axis deviation

Regarding the QRS axis, the left-axis deviation was the most prevalent in both groups. Its prevalence increased with age in both genders, particularly in the > 65 age group. Some studies have reported similar findings and signified that QRS-axis starts rotating to the left in older age groups until it almost becomes flat in a horizontal position after 70 years of age [8, 38, 39]. Wu et al. found that the QRS axis degree in both genders starts decreasing with age, shifting to the left by almost 25°, and reaches a low of approximately 44° after 65 years old [39]. Consistent with our study, Mirahmadizadeh et al. measured a higher mean QRS axis degree among males compared to females [34], which was in line with our results.

### ECG abnormalities

Generally, the older age groups had a higher prevalence of AF, AFL, LBBB, RBBB, sinus bradycardia, PVC, PAC, first-degree AV block, incomplete RBBB, incomplete LBBB, and LAFB. When comparing genders, the prevalence of ECG abnormalities in two groups of males and females did not follow a similar trend. Most ECG abnormalities, such as AFL, RBBB, WPW, sinus bradycardia, PVC, PAC, first-degree AV block, incomplete RBBB, LAFB, and LPFB, had a statistically significant or non-significant higher prevalence among males. Conversely, AF and LBBB were more prevalent among females, although these differences were not significant.

### Major abnormalities

Major ECG abnormalities were observed in 2.9% of the total study population and were more common among men than women. RBBB was the only major abnormality with a statistically significant difference between the genders. Several other studies have reported a comparatively higher prevalence of major abnormalities among men [37, 40–42], whereas few have stated the opposite [43]. De Bacquer et al. measured a significant male-to-female ratio of 1.66 for major ECG abnormalities in their study sample [41]. Two studies conducted in the USA, the Charleston [44] and the Evans heart study [45], reported a prevalence of approximately 7% for major ECG findings in middle-aged men free of coronary heart disease. These discrepancies in the incidence of major abnormalities could be interpreted due to differences in several factors, including ethnicity, sampling methods, socioeconomic status, and ECG definitions and classifications.

In addition, the odds of having major ECG changes significantly increased in both genders with aging (Fig. 2). Our findings were consistent with several other studies indicating the association of age with major ECG abnormalities [37, 40]. Denes et al. also signified that in both men and women, the odds of major ECG abnormalities significantly increased with age [40]. In our study, increased age had a stronger association with major ECG abnormalities among men compared to women.

### AF and AFL

According to the Global Health Data Exchange database, the prevalence of AF was estimated at 0.51% of the world population, with males being affected more than females [46]. Few studies are available on the prevalence of arrhythmia in Iran or the Tehran population. In our study, the prevalence of AF was estimated to be 0.52%, consistent with large multinational studies. Moreover, the AF prevalence remained almost unchanged in both genders until age 55, but it rapidly increased among women after 55 and men after 65 years of age. Several previous studies have also reported an AF prevalence rate ranging between 0.33 to 0.95% and increasing rates with age, specifically after 65 years [37, 38, 47–50]. One study among approximately 3 million participants reported that the absolute prevalence of AF in both genders was almost equal. At the same time, 60% of AF cases in the > 75-year-old age group belonged to women [48]. Surprisingly, in our study, AF was more common among women. Although this was statistically insignificant, many studies reported higher AF rates among males [38, 40].

### Complete bundle branch blocks (both LBBB and RBBB)

Generally, the prevalence of RBBB was higher compared to LBBB, and RBBB was significantly more common among men, while on the contrary, LBBB was more observed among females. Moreover, BBBs prevalence increased substantially with age, specifically in the > 65 groups. Two studies conducted in northern Europe with large populations also had similar findings, manifesting that RBBB was associated with aging and was more prevalent in males [38, 51]. Also, Rodríguez et al. showed that among 13,179 Spanish workers, the complete RBBB and complete LBBB were present in 1.1, and 0.2% of the participants, respectively. One cohort study reported a prevalence of 3.2% for complete RBBB and found that male sex and age were associated with its incidence. They also claimed that bi-fascicular block (BFB) was significantly associated with all-cause mortality [52]. Eriksson et al. followed male participants for 30 years and found a significant surge in BBBs prevalence from 1% at age 50 to 17% at age 80 [53]. Regarding LBBB, Jeong et al. found that incidences of complete LBBB were 0.1% and 0.3% in > 40 and > 65 age groups, respectively, and reported that 71.4% of LBBB cases had more than 65 years of age [54]. Finally, by enrolling 17,489 participants, Hardarson et al. concluded that LBBB prevalence was 0.43% for men and 0.28% for women. The prognosis of LBBB in this study was relatively benign, as few patients required pacemakers [55].

### Minor abnormalities

The prevalence of sinus bradycardia, PVC, PAC, first-degree AV block, incomplete RBBB, incomplete LBBB, and LAFB increased with age. Generally, minor abnormalities were more prevalent among men compared to women. Yu et al. investigated 13,983 cases and demonstrated minor ECG problems in 9.92% of participants. In the 20–44, 45–59, and ≥ 60 age groups, 11.05, 10.82, and 14.26% of men and 6.58, 7.85, and 14.17% of women suffered from minor arrhythmia, respectively [42]. In addition, in De Bacquer et al. study, the prevalence of minor ECG alterations was slightly higher among men (10.4%) compared to women (9.5%) [41]. These discrepancies in minor ECG abnormalities could be due to racial differences, different ECG abnormalities definitions, and various sample sizes.

### Premature contractions (PAC and PVC)

The incidence of both PVC and PAC abnormalities in our study increased significantly with aging, specifically in the > 65-year-old group. Both abnormalities were higher among males, with PVC being substantially more prevalent. Previous studies using standard 12-lead ECG also reported a PVC prevalence rate of 1 to 4%. Interestingly, this number reaches 99% in Holter monitoring studies, with males being affected more than women [40, 56–58]. Similarly, Amir et al. reported a prevalence of 1.1% for PVC and, surprisingly, a relatively higher PVC incidence in females [59]. In another study with the administration of Holter monitoring, increasing numbers of PACs per hour with aging were demonstrated [58]. Also, using a 10-s ECG, a significantly higher prevalence of PAC has been reported at 8% among 63,197 participants between 40 to 79 years old in a Japanese cohort [60]. Thus, the detection methods, age, gender, and ethnicity can play an essential role and might explain the variability of PVC and PAC prevalences in different studies.

### Fascicular hemi-blocks (LAFB, and LPFB)

In our study, LAFB was significantly higher among men than women. Studies reported considerable differences regarding the prevalence of LAFB in the general population. Krivisky et al. reported a prevalence of 1.03% [61]. A prevalence of 6.2% in 3933 participants has also been indicated by Rabkin et al. [62]. These discrepancies can be attributed to ethnic diversities or different underlying pathologies. According to the American heart association (AHA), the prevalence of LAFB in the normal population ranges between 0.9% to 6.2% [63]. In addition, LPFB was seen in 0.41% of our study population. Previous studies have noted that its isolated incidence is infrequent in the general population and is invariably associated with RBBB in some patients with cardiac diseases [63, 64].

### Brugada, and early repolarization

In our study, Brugada Pattern, anterior early repolarization, and inferior/inferolateral early repolarization were significantly more prevalent in males. Almost all cases bearing the Brugada pattern in our study were males. Although the overall prevalence of inferior/inferolateral early repolarization decreased significantly with aging, the other two patterns mostly did not change. A meta-analysis of 29 articles assessed the prevalence of early repolarization at 11.6%. It also indicated that the incidence of early repolarization was 17.0% in men and 6.2% in women, concluding that early repolarization is highly prevalent in men, Blacks, and physically active individuals [65].

One systematic review of 28 articles estimated that the global pooled prevalence of Brugada was 0.05%, with a higher prevalence among males and a preponderance among residents of Southeast Asia [66].

## Limitations

The study's main limitation was excluding participants who did not meet the criteria or did not respond. However, this limitation was addressed by the study's extensive sample size and the inclusion of participants from all city districts, which helped mitigate this issue. Other patient characteristics, such as lifestyle factors, medications, and comorbidities, might have correlated with our study's ECG findings, requiring further investigation. Moreover, We could not differentiate between normal variations and pathologic ECG findings in this study due to lack of other cardiovascular assessments, such as echocardiography.

## Conclusion

Our data notes that age and sex should be taken into consideration when interpreting basic ECG values. For instance, resting HR was higher among women, whereas other ECG values, such as P-wave, RR interval, and QRSd, were higher among men. In addition, major and minor ECG abnormalities were more prevalent in the male population. Also, the odds of having major ECG abnormalities rose with increased age in both genders.

## Data Availability

The datasets regarding the current study are available from the corresponding author upon reasonable request.

## Abbreviations

AF: Atrial Fibrillation
AFL: Atrial Flutter
ANOVA: Analysis of Variance
AV: Atrioventricular
BBB: Bundle Branch Block
CHB: Complete Heart Block
CI: Confidence Interval
CVDs: Cardiovascular Diseases
DBP: Diastolic Blood Pressure
ECG: Electrocardiography
HDL: High-Density Lipoprotein
HR: Heart Rate
LAFB: Left Anterior Fascicular Block
LDL: Low-Density Lipoprotein
LPFB: Left Posterior Fascicular Block
PAC: Premature Atrial Contraction
PJC: Premature Junctional Contraction
PVC: Premature Ventricular Contraction
QRSd: QRS Duration
RBBB: Right Bundle Branch Block
RR interval: R-R Interval
SBP: Systolic Blood Pressure
TG: Triglycerides
WPW: Wolff-Parkinson-White
